# Water-Triggered Self-Healing Composite Coating: Fabrication and Anti-Corrosion Application

**DOI:** 10.3390/polym14091847

**Published:** 2022-04-30

**Authors:** Zhentao Hao, Si Chen, Zhiwei Chen, Zhifeng Lin, Weihua Li

**Affiliations:** 1 School of Chemical Engineering and Technology, Sun Yat-sen University, Zhuhai 519082, China; haozht3@mail2.sysu.edu.cn (Z.H.); chens6@mail2.sysu.edu.cn (S.C.); chenzhw58@mail2.sysu.edu.cn (Z.C.); 2 Southern Marine Science and Engineering Guangdong Laboratory (Zhuhai), Sun Yat-sen University, Zhuhai 519082, China; 3 School of Materials Science and Engineering, Sun Yat-sen University, Guangzhou 510275, China

**Keywords:** corrosion resistance, smart release, self-healing coating, MOF-199

## Abstract

Self-healing coatings formulated by stimuli-responsive container technology are regarded as a prospective strategy for long-term corrosion protection. However, such types of coatings suffer from low coating adaptability and delays in corrosion protection because the occurrence of corrosion is prior to the release of healants from containers. Herein, we took advantage of the easy hydrolysis of MOF-199 for water-induced self-healing properties. Mixed corrosion inhibitors were loaded into MOF-199 and then incorporated into acrylic coating. The water sensitivity of MOF-199 was investigated and EIS tests were used to evaluate the self-healing performance. Due to the collapse of the porous MOF-199 structure, corrosion inhibitors could be released from MOF-199 with the invasion of water into acrylic coating. The corrosion resistance performance of damaged self-healing coating gradually increased. The metal exposed to artificial defects was well protected due to a barrier formed by corrosion inhibitors. Owing to these merits, this self-healing coating is recommended for use in various fields of engineering for corrosion resistance.

## 1. Introduction

Endowing coating with self-healing functions is considered as a promising strategy for long-term metal corrosion protection. Self-healing coating can heal the microcrack or significantly prolong the life cycle of coatings. In recent years, various strategies have been developed to achieve self-healing effects, including embedment of healant-loaded nanocontainers into the coating [1,2,3], shape memory effect of polymer resin [4], and dynamic bonds inside the polymer coating [5], etc. Among these approaches, incorporating healant-loaded containers into a coating for smart release control has drawn much attention because of its spontaneity and simplicity in the realization of self-healing under mild conditions. Various containers, including *β*-cyclodextrin [6,7], graphene oxide [8,9,10,11], silica [12,13,14], halloysite [8,15], titanium dioxide (TiO_2_) [2], nanotubes porous polystyrene microspheres [3], metal-organic framework (MOF) [16,17], etc. have been used for encapsulation of corrosion inhibitors as well as the smart release of various corrosion inhibitors (benzotriazole [9,18], Ficus racemosa leaf extract [19,20], 8-hydroxyquinoline [21], cerium salt [22], molybdate [23] etc.). These containers can respond to environmental changes caused by corrosion or coating degradation such as pH and release healants, which has notably advanced the development of anti-corrosion self-healing coatings. For example, Chen et al. designed a self-healing coating by embedding the molybdate-loaded TiO_2_ into polypyrrole (PPy) [2]. They found that PPy coating gradually degrades with time. However, the pH change induced by localized corrosion can trigger the release of corrosion inhibitors from TiO_2_ nanocontainers to deter corrosion so defects exposed in corrosion media can be repaired. Therefore, the PPy/Mo@TiO_2_ coatings can serve as an excellent self-healing coating and provide long-term corrosion protection. However, some limitations are exposed as well. First, the occurrence of significant local pH changes is due to the formation of OH^−^ at cathodic areas and the hydrolysis of metallic ions at anodic areas during corrosion reaction. At this time, the metal substrate is severely corroded. This severe corrosion is ahead of the release of corrosion inhibitors. Additionally, since some containers can release corrosion inhibitors in response to pH, they may not be very suitable for coatings whose intrinsic environments are acidic or alkaline. For example, the crosslink of the epoxy oligomer usually needs an alkaline curing agent, making the resultant coating alkaline. Therefore, there is still room for the advancement of self-healing coating to solve these challenges.

Metal-organic frameworks (MOFs) are a kind of crystalline porous structure formed by self-assembly of metal ions and organic ligands with the features of high specific surface area and regular tunnel. MOF-199, also known as HUSKT-1, is a subclass of MOFs formed by copper ion coordination with 1,3,5-benzenetricarboxylic acid (BTC) ligands. MOF-199 is prone to acidolysis and hydrolysis. An investigation of water-triggered MOF to release corrosion inhibitors was implemented in this work because the invasion of corrosion media is accompanied with salt neutral water in marine environments. MOF-199 as a container was used to load the corrosion inhibitor and then they were embedded into the coating. When corrosive media penetrates the coating and contact with MOF, the MOF will decompose, leading to the release of the corrosion inhibitor ahead of the local pH change caused by severe corrosion.

## 2. Materials and Methods

### 2.1. Materials

N, N-Dimethylformamide (DMF), and dichloromethane (CH_2_Cl_2_) were purchased from Shanghai Aladdin Biochemical Technology Co., Ltd. (Shanghai, China). BTC, sodium molybdenum oxide (Mo), and ethanol were obtained from Shanghai Macklin Biochemical Co., Ltd. (Shanghai, China). CuCl_2_·2H_2_O and 1H-Benzotriazole (BTA) were offered by Tianjin Baishi reagent Co., Ltd. (Tianjin, China). Deionized water was obtained from the Millipore purification system. Q235 carbon steel (CS) was obtained from the Xunjin stainless steel processing center (Zhejiang, China). The commercial clear acrylic resin was kindly provided by Liaoning Pengwei Chemical Paint Co., Ltd. (Liaoning, China). All reagents were used as received.

### 2.2. Fabrication of MOF-199

MOF-199 was prepared via a typical hydrothermal method. CuCl_2_·2H_2_O (0.2319 g) and BTC (0.1765 g) were added in a test tube and then ultrasonically dissolved in 9 mL mixed solvent (water:DMF:ethanol = 1:1:1 in volume). The test tube was tightly sealed by a stopper. The solution was heated to 85 °C under vigorous stirring for at least 24 h. The solution turned from green to blue and precipitation gradually was formed with time. The resultant blue precipitation (MOF-199) was collected by filtration at room temperature and rinsed with DMF twice to remove the remaining reactants. Then the MOF-199 was further adequately rinsed with CH_2_Cl_2_ to remove the solvent. The product was finally obtained after drying in vacuum at 60 °C for 24 h. The yield of the obtained MOF-199 was ~80%.

### 2.3. Fabrication of BTA-Mo@MOF-199

BTA (0.1 g) and Mo (0.1 g) were dispersed in 50 mL ethanol. Then MOF-199 (0.1 g) was added into the mixture under vigorous stirring for 24 h. Then the mixture was collected by filtration and rinsed with ethanol to remove the unstable immobilized corrosion inhibitor. The production was obtained after drying at 50 °C for 24 h.

### 2.4. Fabrication of Self-Healing Acrylic Coating

**Figure 1:**
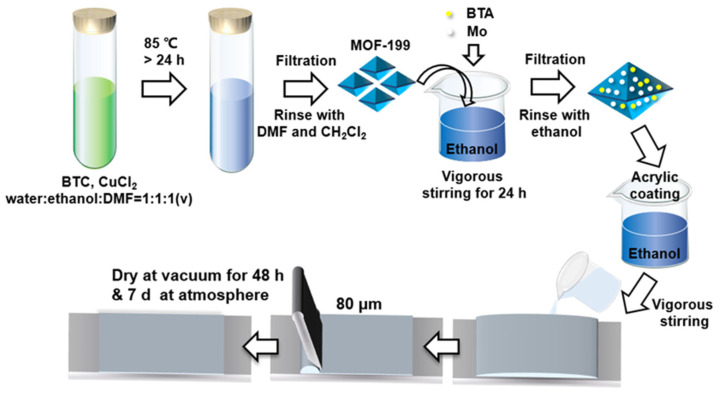
Schematic of the fabrication process of self-healing acrylic coating loaded with BTA-Mo@MOF-199.

BTA-Mo@MOF-199 (0.1 g) and commercial acrylic coating (2 g) were dispersed in ethanol under vigorous stirring. Then the well-dispersed mixture was rapidly coated on carbon steel by an automatic coating applicator (BEVS-1188, BEVS Industrial Co., Ltd. Guangzhou, China). The coating thickness was ~80 μm. To remove the bubble gas inside the coating as possible, the as-prepared sample was placed in a vacuum for 48 h. The composite coating, denoted as BTA-Mo@MOF-199/acrylic coating, was finally obtained after drying at room temperature for 7 days. The whole process is demonstrated in Figure 1.

### 2.5. Characterization

The morphology of the MOF-199 and self-healing coating were observed by Field Scanning Electron Microscope (FSEM, JOEL) and the energy dispersive spectrum (EDS) was mapped for element analysis. The FTIR spectrum of the MOF-199 was measured by an infrared spectrometer (EQUINOX 6000) at the wavelength of 4000–500 cm^−1^. XRD diffraction patterns of all samples were determined by an X-ray diffractometer (Bruker D8 ADVANCE). The thermogravimetric analysis was performed on thermogravimetry (TG209F1 libra). Samples were heated from room temperature to 900 °C at the rate of 10 °C/min in nitrogen. N_2_ adsorption/desorption at liquid nitrogen temperature was used to determine specific surface areas and pore volumes of the samples as well as the pore sizes of samples by Micromeritics ASAP 2460. All samples were outgassed at 343.15 K for 12 h under a vacuum of 10^−4^ Pa before measurements. Electrochemical impedance spectroscopy (EIS) was conducted to investigate self-healing performance of samples by an electrochemical station (Gamry 600) with an amplitude of 20 mV at the frequency range from 10^5^ to 10^−2^ Hz. (For the bare Q235 carbon steel, an amplitude of 2 mV was applied because of the very small resistance). Prior to the self-healing test, samples were damaged to form a scratch with a length of ~2 mm by a scalpel. Then, damaged samples with 1 cm^2^ as working electrode were immersed in 3.5 wt.% NaCl aqueous solution. Pt and calomel electrodes were used as counter and reference electrodes, respectively.

## 3. Results and Discussion

### 3.1. Characterization of the Synthesized MOF-199

**Figure 2:**
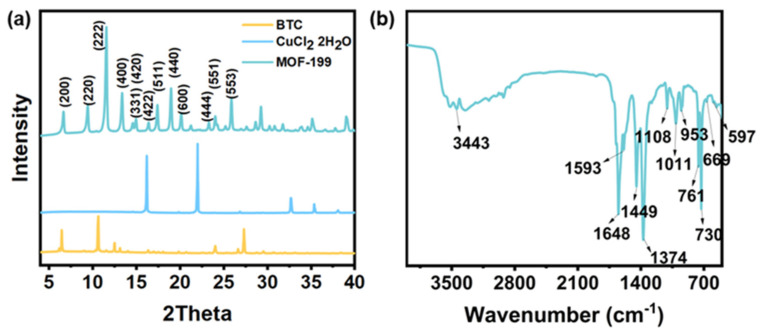
(**a**) XRD patterns of BTC, CuCl_2_ H_2_O, and MOF−199. (**b**) FTIR of MOF−199.

The MOF-199 was synthesized by the reaction of CuCl_2_ H_2_O and BTC in the mixed solvent. XRD and FTIR were used to characterize the resultant MOF-199. The XRD patterns and FTIR spectrum of MOF-199 are indicated in Figure 2. In the XRD spectrum, the crystal structure of the MOF-199 was quite different from reactants (CuCl_2_ H_2_O and BTC), which showed the huge difference in diffraction peaks (Figure 2a). Characteristic peaks of MOF-199 could be found at 2θ = 6.8°, 9.5°,11.8°, 13.4°, 17.5°,and 19.2°. There were no other diffraction peaks found, implying the degree of high purity of the prepared MOF-199, which is in agreement with the previous literature [24]. In the FTIR spectrum (Figure 2b), a broad peak 3500–2700 cm^−1^ was observed in the –OH groups due to the presence of the remaining water in the structure of hydrophilic MOF-199. The vibration of –OH single bonds at 1617 cm^−1^ also revealed that MOF-199 had water. Peaks at 1700–500cm^−1^ are a result of the vibration of the main MOF functional groups [25]. In detail, the peak at 1648 cm^−1^ is the stretching vibration of carboxylate anions reflecting the reaction of copper ions with –COOH groups in the BTC [26]. Characteristic peaks of MOF-199 could be found at 730 and 761cm^−1^ because of metal Cu substitution on benzene groups. The peak at 1094 cm^−1^ is the result of COO- and Cu stretching vibration of MOF. The peaks at 1374 and 1449 cm^−1^ are due to the vibrations of the –COO group in MOF-199 bidentate behavior of COO moiety.

### 3.2. Characterization of the BTA-Mo@MOF-199

**Figure 3:**
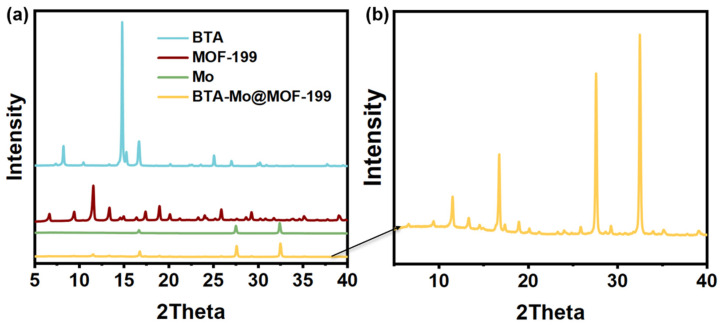
(**a**) XRD patterns of BTA, MOF−199, Mo, BTA−Mo@MOF−199. (**b**) The magnified XRD pattern of BTA-Mo@MOF−199.

The XRD patterns of MOF-199, BTA-Mo@MOF-199, and corrosion inhibitors are indicated in Figure 3. Samples exhibited diffraction peaks of MOF-199 even after corrosion inhibitor incorporation inside their pores, which showed that the prepared MOF-199 is a stable container to encapsulate these corrosion inhibitors. After incorporation of BTA and Mo, the main diffraction peaks of MOF 199 could still be observed at the characteristic peaks of the pure MOF-199 (2θ = 6.8°, 9.5°, 11.8°, 13.4°, 17.5°, and 19.2°). The XRD pattern of the BTA-Mo@MOF-199 was similar to that of MOF-199, reflecting that the incorporation of corrosion inhibitors can be achieved without destroying the MOF-199 structure. Peaks of both Mo (2θ = 16.6°, 27.5°, 32.4°) and BTA (2θ = 8.2°, 10.4°, 14.7°, 15.2°, 16.6°) could also be found in BTA-Mo@MOF-199. Besides, intensities of the peaks of MOF-199 decreased, which can be considered another evidence of the introduction of the BTA and Mo into the pores. Furthermore, there was no apparent loss of crystallinity in X-ray diffraction patterns and there were no supplementary peaks found after introducing the corrosion inhibitors, indicating the high purity of these samples.

**Figure 4:**
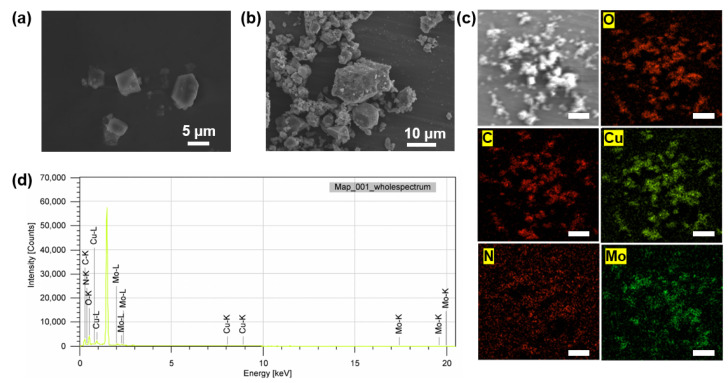
SEM of (**a**) the MOF-199 and (**b**) BTA-Mo@MOF-199. (**c**) EDS mapping of BTA-Mo@MOF-199; the scale bar is 50 μm. (**d**) EDS spectra of BTA-Mo@MOF-199.

The SEM in Figure 4a shows that a crystalline material in the shape of octahedral with a size of 4–7 μm was achieved. The corrosion inhibitor could not be very clearly observed in SEM of BTA-Mo@MOF-199 (Figure 4b) due to the dispersion of corrosion inhibitors at the nanoscale. However, the distribution of these corrosion inhibitors on MOF-199 could be visualized by EDS mapping (Figure 4c). The peaks of N, C, O, Cu, and Mo elements are distinct in Figure 4d, confirming the presence of these elements in BTA-Mo/MOF-199 structures. N and Mo elements belong to the BTA and molybdate, respectively while Cu, O, and C elements are from the MOF-199 structure. The distribution of N and Mo elements is in agreement with the MOF-199 structure, proving the corrosion inhibitor is well incorporated into MOF-199.

### 3.3. Thermostability of MOF-199 and BTA-Mo@MOF-199

**Figure 5:**
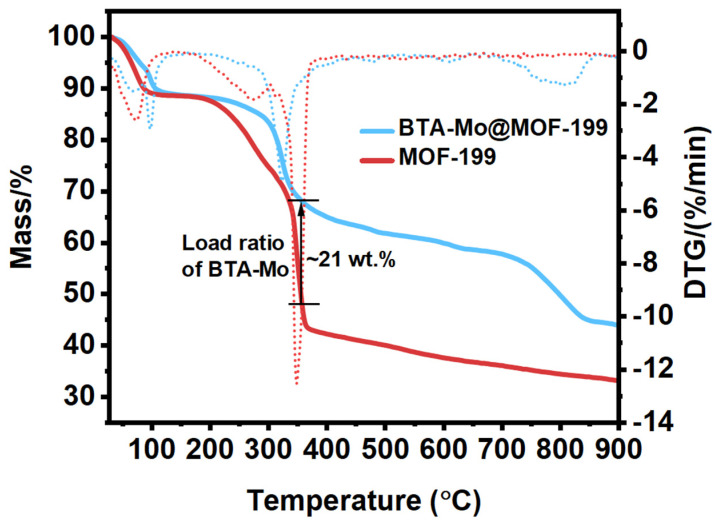
Thermogravimetry analysis of MOF-199 and BTA-Mo@MOF-199.

The thermal stability of MOF-199 is necessary to investigate in practical applications. Figure 5 shows the thermostability of MOF-199 at the temperature range of 25 °C to 900 °C in nitrogen gas. There are three main weight changes observed in the pure MOF-199. With the temperature gradually increasing to ~90 °C, the weight of pure MOF-199 lost ~12% owing to the evaporation of the residual mixed solvents inside pores including water, DMF, and CH_2_Cl_2_. The second main weight loss (45 wt.%) of pure MOF-199 is due to the decomposition of the MOF-199 at the temperature range of 200~380 °C [27]. This also reflects that the MOF-199 can remain stable below 200 °C. The third main 8% weight loss of MOF-199 is due to the evaporation of the product after decomposition of MOF-199. The residual weight of MOF-199 at a higher temperature may be related to copper salt. A similar weight loss of BTA-Mo@MOF-199 at ~90 °C could be also found. The second main weight loss of ~23% occurred between 200 °C and 380 °C, which is lower than pure MOF-199 due to the presence of corrosion inhibitors (BTA and Mo). After decomposition of MOF-199, the mass difference between pure MOF-199 and BTA-Mo@MOF-199 was ~21 wt.% at 350 °C. So, it can be deduced that the mass ratio of BTA-Mo accounts for ~21 wt.%. The third main weight loss of 13.35% occurred between 380 °C and 900 °C due to the evaporation of the products after decomposition.

### 3.4. Water Sensitivity of MOF-199

**Figure 6:**
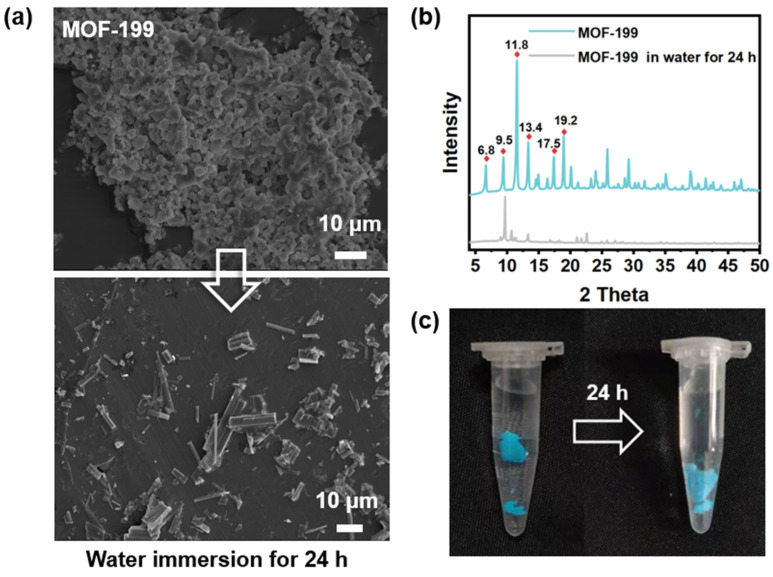
(**a**) SEM, (**b**) XRD patterns, and (**c**) images of MOF-199 before and after being immersed in water for 24 h.

To investigate the water sensitivity of MOF-199, we immersed MOF-199 into water and made a comparison in MOF structure. Figure 6 presents a comparison of MOF-199 before and after water immersion. The morphology of the MOF structure evolves to a rectangular shape as shown in Figure 6a. The intensity of all peaks has significant decrease and many characteristic peaks (2θ = 6.8°, 9.5°,11.8°, 13.4°, 17.5°, and 19.2°) of MOF-199 are lost in the XRD pattern after 24-h water immersion (Figure 6b). The hydrolysis reaction of MOF-199 is as follows:

Cu_3_(BTC)_2_+ 6H_2_O = 3Cu(OH)_2_ + 2H_3_BTC

**Figure 7:**
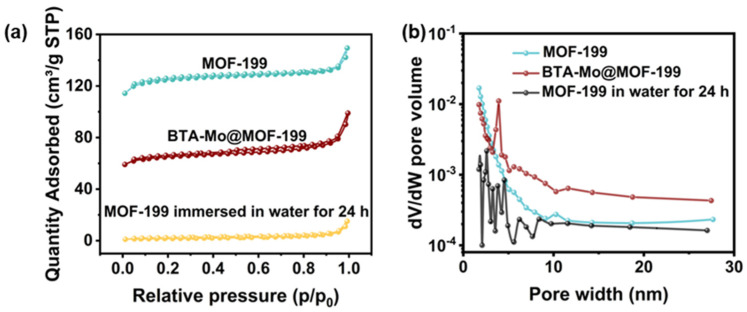
(**a**) BET adsorption−desorption curve and (**b**) pore−sized distribution of MOF−199, BTA−Mo@MOF−199, and MOF−199 in water for 24 h.

Furthermore, the surface area and pore size distribution of MOF-199, BTA-Mo@MOF-199, and MOF-199 in the water immersion test were also compared as shown in Figure 7. MOF-199 and BTA-Mo@MOF-199 presented a mixture of both type I and type IV N_2_ adsorption-desorption isotherms according to IUPAC classification (Figure 7a). The feature of pure MOF-199 in the adsorption-desorption curve is the rapid rise of adsorption capacity at a lower relative pressure, and saturation appears when the adsorption reaches a certain relative pressure. This is due to the presence of the microporous, in agreement with the pore size distribution in Figure 7b. Specific surface area and average pore volume determined from N_2_ adsorption-desorption isotherms are listed in Table 1. In fact, many factors such as the preparation method, activation temperature, and small solvent molecules remaining in the porous structure will significantly affect the surface area. In this work, Langmuir surface area and pore volume of pure MOF-199 were found to be 598.75 m^2^/g and 0.21 cm^3^/g, respectively. Furthermore, after encapsulating corrosion inhibitors, the values of surface area and the average pore volumes of MOF 199 were significantly decreased to 341.39 m^2^/g and 0.12 cm^2^/g, respectively, compared with the pure MOF 199 (Table 1). This is because the porous structure is occupied by the corrosion inhibitor, leading to blocking of some pores and decreased accessibility of N_2_ gas to pores. This can also suggest the incorporation of corrosion inhibitors inside the MOF 199 pores. Moreover, it is worth noting that both surface area and pore volume sharply decreased to 31.65 m^2^/g and 0.01 cm^3^/g, respectively, after immersion in water for 24 h. This indicates the adsorption capacity of MOF-199 immersed in water was largely reduced because the structure of the MOF-199 was changed. The water stability of MOF-199 is determined by the battle between the BTC ligands and water molecules. Pore structure collapse of MOF-199 is caused by the coordination between the water and the Cu^2+^ metal center and displacement of the BTC ligand in MOF-199 [22], which can be revealed by the phenomenon that MOF-199 turns white after 24 h immersion in water (Figure 6c). This also demonstrates that MOF-199 can also serve as a water indicator. Since the collapsed MOF-199 structure has a limited surface area and absorbance capacity, the corrosion inhibitor encapsulated by MOF-199 will be rapidly released due to the structure collapse after in contact with water molecules. This lays the basis for the smart release of corrosion inhibitors from containers triggered by water for self-healing coating.

**Figure 8:**
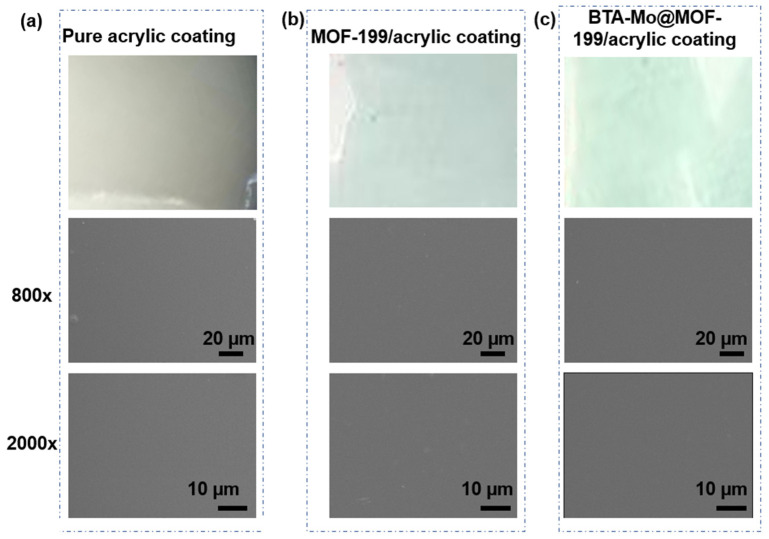
Optical images and SEM with 800× and 2000× magnification of (**a**) pure acrylic coating, (**b**) MOF-199/acrylic coating, and (**c**) BTA-Mo@MOF-199 acrylic coating.

Figure 8 compares the surface morphology of the coating samples. The pure acrylic coating showed a smooth and glossy surface. It can be found that the pure coating presented an intact surface without defects such as cracks and pits in the SEM image. After MOF-199 or BTA-Mo@MOF-199 was introduced into the acrylic coating, the color of the acrylic coating changed from transparent to blue-green (Figure 8b). The optical image of BTA-Mo@MOF-199/acrylic coating was similar to that of MOF-199/acrylic coating. Besides, the uniform color distribution of the coating means that MOF-199 and BTA-Mo@MOF-199 were well dispersed. Furthermore, all of the coating samples showed a similar morphology in SEM. There were no observable cracks or pits in SEMs of MOF-199/acrylic and BTA-Mo@MOF-199 coatings. Therefore, the introduction of MOF-199 or BTA-Mo@MOF-199 did not bring significant defects to the coating surface, which is beneficial for coatings in the application of corrosion protection in marine environments.

### 3.5. Self-Healing Performance of Acrylic Coating Doped with BTA-Mo@MOF-199

**Figure 9:**
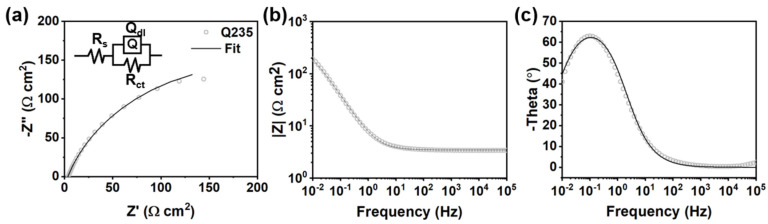
(**a**) Nyquist plot and corresponding equivalent circuit of bare Q235 carbon steel. (**b**) Bode−modulus and (**c**) Bode−phase plots of bare carbon steel.

The self-healing coating was achieved by introducing BTA-Mo@MOF-199 into the commercial acrylic coating. To verify the availability of self-healing property, coating samples were artificially scratched by a scalpel, and EIS was conducted by immersion of damaged coatings in 3.5 wt.% NaCl with time. The damaged pure acrylic and MOF-199/acrylic coatings served as control groups. In EIS, the |Z| modulus at the lowest frequency (|Z|_0.01Hz_) can also indicate the overall resistance in the electrochemical system for a semi-qualitative analysis of the corrosion resistance. Large |Z|_0.01Hz_ means high resistance in the electrochemical system. For the bare carbon steel, a small semicircle and a low |Z|_0.01Hz_ can be observed in Nyquist and Bode plots (Figure 9a,b), which means freshly polished carbon steel is extremely susceptible to corrosion. The one-time constant detected in the Bode-phase plot reflects only one capacitance phase element (Q) involved in an equivalent circuit (Figure 9c). Therefore, the model R(QR) is used to fit the EIS plot of carbon steel. These fit parameters are listed in Table 2. R_s_, R_f_, and R_ct_ refer to the resistance of NaCl aqueous solution, acrylic coating, and charge transfer resistances, respectively. The capacitance phase element is used to replace the ideal capacitance. Q_dl_ and Q_f_ refer to the double-layer capacitance and capacitance of the coating, respectively. The impedance of Q can be expressed as [28,29]:

$$Z_{Q}={\;Y}_{0}^{-1}{\left(j\omega \right)}^{-n}$$

where Y_0_, j, ω, and n refer to Q coefficient, imaginary unit, angular frequency, and exponent reflecting deviations from ideal capacitance caused by the roughness or surface defects, respectively.

**Figure 10:**
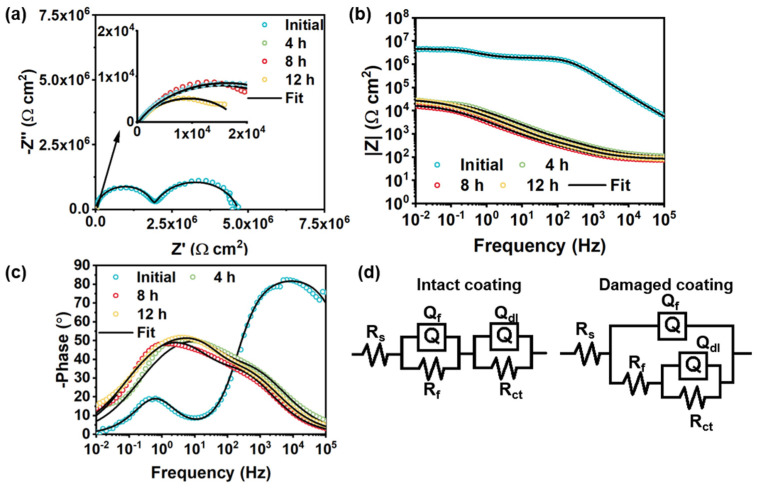
Evolution of (**a**) Nyquist plots, (**b**) Bode−modulus plots, and (**c**) Bode−phase plots of damaged pure acrylic coating with time. (**d**) Equivalent circuit of the intact and damaged pure acrylic coating.

**Figure 11:**
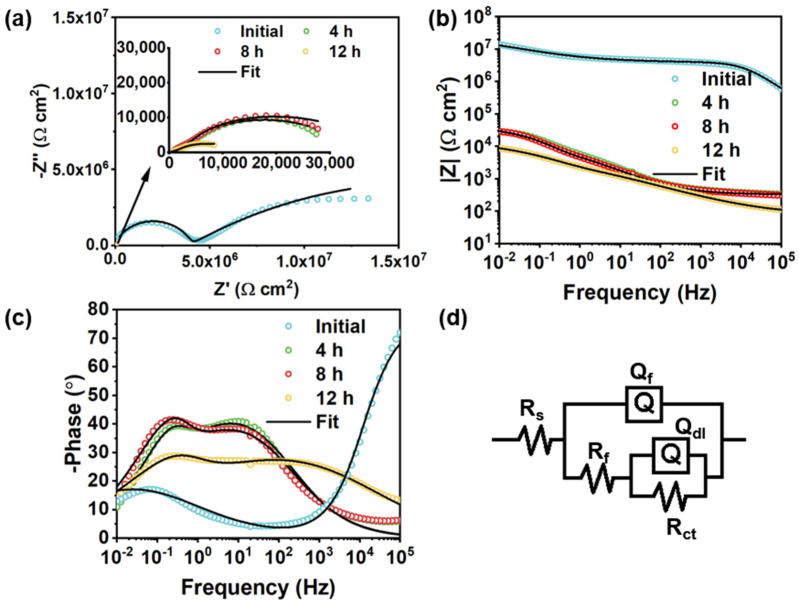
Evolution of (**a**) Nyquist plots, (**b**) Bode−modulus plots, and (**c**) Bode−phase plots of damaged MOF−199/acrylic coating with time. (**d**) Equivalent circuit of the intact and damaged MOF−199/acrylic coating.

In the control group, the semicircle radius of the damaged pure acrylic coating showed a significant decrease as shown in Figure 10a,b. This is similar to the MOF-199/acrylic coating (Figure 11a,b). The |Z|_0_._01Hz_ of the damaged pure acrylic coating decreased to ~1.6 × 10^4^ Ω cm^2^ in 12 h, compared with the original intact one (~4.6 × 10^6^ Ω cm^2^) (Figure 10b). For the pure acrylic coating without fillers, a continuous phase of resin is presented in the coating, making the coating defectless, and the two-time constant can be observed in Figure 10c. Therefore, the model R(QR)(QR) was used to fit the EIS plot of the intact pure acrylic coating. After the coating was damaged, the presence of the coating defect shifted the intact fit model to R(Q(R(QR))). For the MOF-199/acrylic coating and BTA-Mo@MOF-199/acrylic coating, the introduction of MOF-199 or BTA-Mo@MOF-199 makes the phase of acrylic resin discontinuous. So, R(Q(R(QR))) was applied for both intact and damaged of these samples (Figure 11d). Coating capacitance is commonly used to evaluate the coating permeability. Low Q_f_ means low coating permeability. Q_f_ of the damaged pure acrylic coating increased to 47 μF/cm^2^ compared with that of the intact one (6.28 × 10^−4^ μF/cm^2^) in Table 2, suggesting the coating was penetrated by water. Therefore, R_ct_ showed a continuous downward trend with time due to the gradual diffusion of aqueous NaCl to the metal interface. Similar to pure acrylic coating, MOF-199/acrylic coating without corrosion inhibitors also fails with the increase of R_ct_ over time. On the contrary, it significantly decreases with time, demonstrating that the pure MOF-199 had no contribution to the self-healing effect (Table 3). This evidences that pure acrylic coating and MOF-199/acrylic coating cannot achieve self-healing properties.

**Figure 12:**
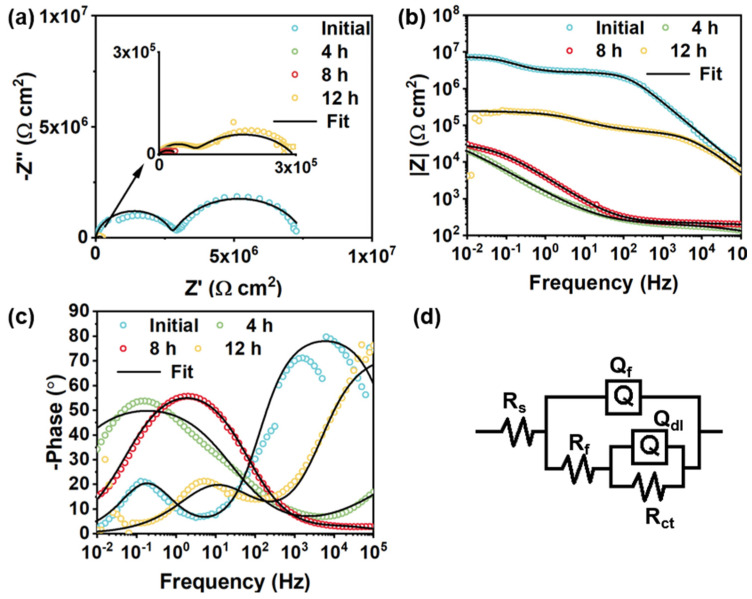
Evolution of (**a**) Nyquist plots, (**b**) Bode-modulus plots, and (**c**) Bode-phase plots of damaged BTA−Mo@MOF−199/acrylic coating with time. (**d**) Equivalent circuit of the intact and damaged BTA−Mo@MOF−199/acrylic coating.

**Figure 13:**
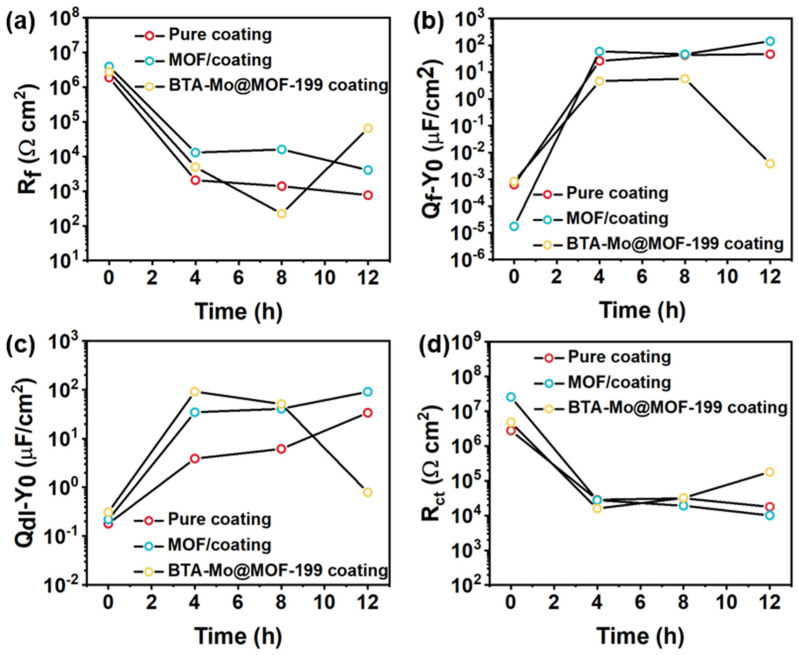
Evolution of (**a**) R_f_, (**b**) Q_f_, (**c**) Q_dl_, and (**d**) R_ct_ with time among pure coating, MOF/coating, and BTA−Mo@MOF−199 coating systems.

**Figure 14:**
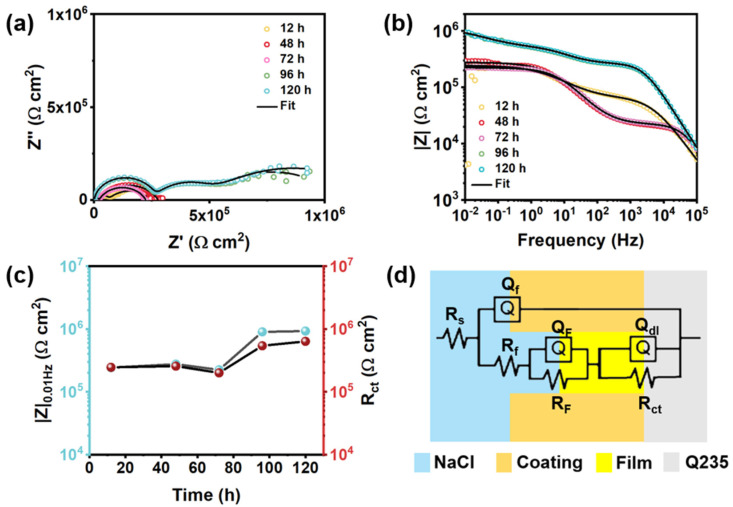
(**a**) Nyquist, (**b**) bode plots, and (**c**) R_ct_ and |Z|_0.01Hz_ of damaged BTA−Mo@MOF−199 coating with time after 12 h; (**d**) Equivalent circuit of damaged BTA−Mo@MOF−199/acrylic coating after 96 h.

**Figure 15:**
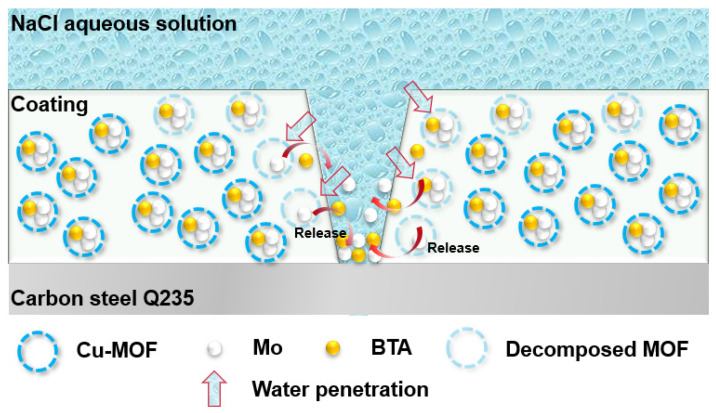
The protection mechanism of the BTA−Mo@MOF−199/acrylic coating.

Figure 12 presents the EIS plots and equivalent circuits of intact and damaged BTA-Mo@MOF-199/acrylic coatings. A significant decrease followed by an increase of semicircle radius and |Z|_0.01Hz_ was observed with time, indicating that the impedance of the whole electrochemical system was enhanced with time after being damaged (Figure 12a). In the EIS measurements of the BTA-Mo@MOF-199/acrylic coating system, within the first 4 h, the |Z|_0.01Hz_ of the damaged sample largely decreased compared with the initial intact coating. However, subsequently, it significantly increased with time (Figure 12b), showing a decreasing trend followed by an increasing trend in overall resistance of the coating system. Likewise, equivalent circuits were proposed to understand the development of the damaged coatings. Since a two-time constant can be found in Figure 12c, R(Q(R(QR)) were used to fit EIS plots of the initial coating (Figure 12d). Fitted parameters are listed in Table 4. In the damaged coating, with the decomposition of MOF-199 induced by water invasion, corrosion inhibitors could be released from MOF-199 and adsorbed on metal surface in 8 h, which was reflected by a slight increase in |Z|_0.01Hz_ shown in Figure 12b. The decline of R_ct_ in 4 h is attributed to the attack of corrosion media on the metal surface. Though corrosion inhibitors were released, the reactions between corrosion productions and corrosion inhibitors were insufficient to form an intact film for the protection of the exposed metal area at this time. However, a significant increase of R_ct_ was observed in 12 h (Figure 13d). A significant Q_dl_ reduction implies the adsorption of a film on the surface and corrosion media detachment, which means corrosion-active centers on the exposed carbon steel surface declined with immersion time (Figure 13c). Due to the formation of a film on the exposed metal area, the diffusion of corrosive media in NaCl aqueous solution, as well as corrosion production on the metal surface, are limited. Compared with other pH-sensitive containers, the use of water-sensitive MOF-199 showed a shorter time to release the corrosion inhibitors, allowing faster repair of defects and protection of exposed metal (Table 5). This is meaningful for application in marine environments. Figure 13 summarizes the electroparameter developments of these samples. The R_f_ of pure acrylic and MOF-199-incorporated coatings decreased with time after being damaged (Figure 13a), resulting in increases in coating permeability (Q_f_) and corrosion reactions (Q_dl_) and decreases in R_ct_. By comparison, these parameters variation of BTA-Mo@MOF-199-incorporated coating is similar to pure acrylic and MOF-199-incorporated coatings before 8 h. However, significant decreases in coating permeability (Q_f_) and corrosion reactions (Q_dl_), as well as increases in R_ct_ were observed in 12 h. Figure 14a presents Nyquist plots of damaged self-healing coatings after 12 h. The semicircle radius of the damaged self-healing coating gradually increased. Additionally, gradual increases in |Z|_0_._01Hz_with time can be observed in Figure 14b. After 96 h, due to the presence of intact film formed by corrosion inhibitors on coating the damaged area, the model R(Q(R(QR)(QR))) was selected to fit the EIS plots (Figure 14d). R_F_ and Q_F_ refer to the resistance and capacitance of film formed by corrosion inhibitors, respectively. R_ct_ can maintain 10^5^ orders of magnitude with time (Figure 14c), reflecting that the film on the damaged coating area can remain stable in corrosive environments. In summary, when the BTA-Mo@MOF-199 coating system was mechanically scratched, some BTA-Mo@MOF-199 remained around the damaged region. In the initial stage, the corrosion resistance of the coating will be depressed, resulting in attack of the metal surface by corrosive media in aqueous solution. Meanwhile, water penetrates through the damaged area and triggers the release of corrosion inhibitor (BTA and Mo) from MOF-199 as illustrated in Figure 15. Then, Mo enables the transformation of γ-FeOOH to Fe_2_O_3_. Then, a dense film containing Fe_2_O_3_, MoO_3_, and FeMnO_4_ was formed on the metal surface. Groups with unpaired electrons in BTA can coordinate with corrosion production (Fe^2+^) to form another film adsorbed on the metal surface. These could depress corrosive media to attack the metal so as to prohibit the further development of the metal corrosion after 12 h.

## 4. Conclusions

In this work, we synthesized MOF-199 via a hydrothermal reaction of BTC and copper salt. The MOF-199 structure with a high pore volume as well as absorbance capacity can serve as a competent container to load the mixed corrosion inhibitors. The MOF-199 exhibited a certain thermal stability and its decomposition temperature reached 200 °C. The MOF-199 structure is prone to collapse in contact with water due to the metal substitution and the decomposed products showing limited absorbance capacity. So, the BTA-Mo@MOF-199 can rapidly release corrosion inhibitors under water stimuli. After the incorporation of BTA-Mo@MOF-199, the corrosion resistance performance of the damaged acrylic coating will gradually increase with time. In summary, the BTA-Mo@MOF-199 can endow the commercial acrylic coating with water detection and self-healing functions. All raw materials used in the facile fabrication of this self-healing acrylic coating are economically accessible. We believe this type of coating is a promising coating in mass production at the plant level and practical corrosion protection in various engineering fields.

## Figures and Tables

**Table 1:** BET parameters of MOF-199, BTA-Mo@MOF-199, and MOF-199 in water.

	MOF-199	BTA-Mo@MOF-199	MOF-199 in Water
Langmuir specific area (m^2^/g)	598.75	341.39	31.65
Pore Volume (cm^3^/g)	0.21	0.12	0.01
Average pore size (nm)	2.20	2.05	7.38

**Table 2:** Electroparameters of the bare CS, intact acrylic coating, and damaged pure acrylic coating with time.

	Bare CS	Initial Intact Coating	Damaged Pure Coating		
			4 h	8 h	12 h
R_f_ (Ω cm^2^)	\	1.89 × 10^6^	2077	1395	768.8
Q_f_−Y_0_ (μF/cm^2^)	\	6.28 × 10^−4^	26.42	43.55	47
Q_f_−n	\	0.94	0.59	0.60	0.63
R_ct_ (Ω cm^2^)	390.4	2.78 × 10^6^	2.85 × 10^4^	3.13 × 10^4^	1.78 × 10^4^
Q_dl_−Y_0_ (μF/cm^2^)	3.75 × 10^4^	0.18	3.89	6.13	33.88
Q_dl_−n	0.80	0.82	0.80	0.82	0.7

**Table 3:** Electroparameters of the bare CS, intact MOF-199/acrylic coating, and damaged MOF-199/acrylic coating with time.

	Bare CS	Initial Intact Coating	Damaged MOF-199/Acrylic Coating		
			4 h	8 h	12 h
R_f_ (Ω cm^2^)	\	3.95 × 10^6^	12,980	16,060	4605
Q_f_−Y_0_ (μF/cm^2^)	\	1.75 × 10^−5^	59.7	46.93	143.5
Q_f_−n	\	0.85	0.56	0.58	0.39
R_ct_(Ω cm^2^)	390.4	2.58 × 10^7^	27,560	19,130	10,070
Q_dl_−Y_0_ (μF/cm^2^)	37,500	0.22	34.72	40.76	91.36
Q_dl_−n	0.8	0.4	1	1	0.74

**Table 4:** Electroparameters of the bare CS, intact self-healing coating, and damaged self-healing coating with time.

	Bare CS	Initial Intact Coating	Damaged Self-Healing Coating		
			4 h	8 h	12 h
R_f_ (Ω cm^2^)	\	2.77 × 10^6^	4.97 × 10^3^	2.25 × 10^2^	2.35 × 10^3^
Q_f_−Y_0_ (μF/cm^2^)	\	8.32 × 10^−4^	4.64	5.69	7.93
Q_f_−n	\	0.91	0.46	0.35	0.44
R_ct_(Ω cm^2^)	390.4	4.82 × 10^6^	1.59 × 10^4^	3.22 × 10^4^	2.45 × 10^5^
Q_dl_−Y_0_ (μF/cm^2^)	3.75 × 10^4^	0.31	91.85	51.24	2.17
Q_dl_−n	0.8	0.8	0.44	0.72	0.7

**Table 5:** Comparison of self-healing coatings by containers technology among different works.

Stimulus	Container	Response Time	Inhibitor	Ref.
pH	TiO_2_	20 d	Mo	[2]
pH	Graphene@ polyaniline	14 d	BTA	[9]
pH	Halloysite nanotubes	3 d	Imidazole	[15]
pH	ZIF-67	30 d	3-Aminopropyl Triethoxysilane	[30]
pH	Zeolitic imidazole framework	5 d	Benzotriazole	[31]
pH	Polypyrrole	10 d	Quaternized alkyl Pyridine	[32]
pH	Halloysite nanotubes	2.5 d	Hydroxyquinoline	[33]
water	MOF−199	12 h	BTA and Mo	This work

## Data Availability

Data is contained within the article.
